# Causes, Patterns, and Severity of Androgen Excess in 1205 Consecutively Recruited Women

**DOI:** 10.1210/jc.2017-02426

**Published:** 2018-01-12

**Authors:** Yasir S Elhassan, Jan Idkowiak, Karen Smith, Miriam Asia, Helena Gleeson, Rachel Webster, Wiebke Arlt, Michael W O’Reilly

**Affiliations:** 1 Institute of Metabolism and Systems Research, University of Birmingham, Birmingham, United Kingdom; 2 Centre for Endocrinology, Diabetes and Metabolism, Birmingham Health Partners, Birmingham, United Kingdom; 3 Department of Clinical Biochemistry, University Hospitals Birmingham NHS Foundation Trust, Birmingham, United Kingdom

## Abstract

**Context:**

Androgen excess in women is predominantly due to underlying polycystic ovary syndrome (PCOS). However, there is a lack of clarity regarding patterns and severity of androgen excess that should be considered predictive of non-PCOS pathology.

**Objective:**

We examined the diagnostic utility of simultaneous measurement of serum dehydroepiandrosterone sulfate (DHEAS), androstenedione (A4), and testosterone (T) to delineate biochemical signatures and cutoffs predictive of non-PCOS disorders in women with androgen excess.

**Design:**

Retrospective review of all women undergoing serum androgen measurement at a large tertiary referral center between 2012 and 2016. Serum A4 and T were measured by tandem mass spectrometry and DHEAS by immunoassay. Patients with at least one increased serum androgen underwent phenotyping by clinical notes review.

**Results:**

In 1205 women, DHEAS, A4, and T were measured simultaneously. PCOS was the most common diagnosis in premenopausal (89%) and postmenopausal women (29%). A4 was increased in all adrenocortical carcinoma (ACC) cases (n = 15) and T in all ovarian hyperthecosis (OHT) cases (n = 7); all but one case of congenital adrenal hyperplasia (CAH; n = 18) were identified by increased levels of A4 and/or T. In premenopausal women, CAH was a prevalent cause of severe A4 (59%) and T (43%) excess; severe DHEAS excess was predominantly due to PCOS (80%). In postmenopausal women, all cases of severe DHEAS and A4 excess were caused by ACC and severe T excess equally by ACC and OHT.

**Conclusions:**

Pattern and severity of androgen excess are important predictors of non-PCOS pathology and may be used to guide further investigations as appropriate.

## Teaser

We studied causes, patterns, and severity of androgen excess in 1205 women at our institution over 5 years and found distinct patterns predictive of non-PCOS pathology in pre- and postmenopausal women.

Clinical or biochemical features of androgen excess are observed in up to 10% of women of reproductive age (1). Androgen excess manifests clinically with hirsutism, androgenic alopecia, acne, ovulatory dysfunction and, if extreme and prolonged, can lead to overt and severe masculinization (2). Recent data have also highlighted the lifelong adverse metabolic consequences of chronic androgen excess in women (3, 4). The overwhelming majority of women presenting with androgen excess have an underlying diagnosis of polycystic ovary syndrome (PCOS) (5). However, complex ovarian, adrenal, and pituitary disorders, including neoplasia, must be excluded, particularly in women with more severe androgen excess and relatively rapid onset of symptoms (6). Detailed clinical history, including acuity of symptom onset, physical examination, biochemical phenotyping, and, where appropriate, radiological investigations are critical in the investigation of those women deemed more likely to harbor more sinister underlying pathology (7). However, there is a distinct lack of data delineating severity and patterns of androgen excess considered predictive of non-PCOS–related disease.

Testosterone (T) and the even more potent 5*α*-dihydrotestosterone (DHT) are biologically active androgens derived from the androgen precursors dehydroepiandrosterone (DHEA) and androstenedione (A4). In women, DHEA, its sulfate ester DHEAS, and its downstream product A4 are produced in large quantities by the zona reticularis of the adrenal cortex (8) and activated to potent androgens in the ovaries but importantly also in peripheral target tissues of androgen action, such as adipose tissue (9). Primary adrenal insufficiency is associated with severe deficiency, not only of DHEA and A4 but also of active androgens (10, 11). Although T can be produced in the adrenal cortex (12) and peripheral tissues, the ovaries are a key contributor to T synthesis, with oophorectomy leading to a major drop in circulating T (13). Therefore, serum T is traditionally regarded as a biomarker of ovarian androgen production (13–15). Conversely, DHEAS is thought to be a marker of adrenal androgen secretion, whereas there is ongoing debate on whether ovary or adrenals are the major source of A4.

Physiologically low circulating T in women, coupled with the widespread use of immunoassays for its quantification, limit the diagnostic utility of serum T in the investigation of hyperandrogenism when measured in isolation (16). Immunoassays are blighted by marked cross-reactivity (17, 18). Liquid chromatography-tandem mass spectrometry has emerged as a highly sensitive and specific tool in biochemical steroid analysis, particularly in the evaluation of very low abundance steroids such as serum androgen concentrations in women, children, and hypogonadal men (19, 20). We have already demonstrated the diagnostic utility of measuring A4 alongside T using mass spectrometry–based techniques in women with PCOS (4). However, mass spectrometry also comes with the capability to measure all androgens simultaneously in a single run, and this can create challenges when confronted with abnormal results for analytes that were not specifically requested.

In this study, we analyzed serum androgen data measured in a large cohort of women consecutively recruited in a United Kingdom secondary/tertiary endocrine referral center over a period of 5 years. Our aims were to gain insights into the diagnostic utility of simultaneous measurement of serum DHEAS, A4, and T in women with suspected androgen excess, to delineate the biochemical signature of underlying diagnoses, and to define serum cutoff levels that may be predictive of underlying non-PCOS pathology.

## Methods

### Subjects and clinical protocol

We included all women who had undergone measurements of serum DHEAS, A4, and T as part of routine clinical care at the University Hospital Birmingham NHS Foundation Trust between 1 January 2012 and 31 December 2016. Institutional review board approval for retrospective data review from patients undergoing routine clinical care was obtained from University Hospital Birmingham (reference CARMS-13345). For the purposes of analysis, we defined women younger and older than 50 years as premenopausal and postmenopausal, respectively, as the average age of menopause in the UK is 51 years. From the cohort of patients with simultaneous measurement of serum DHEAS, A4, and T, we identified a patient subset defined by at least one of the three serum androgens increased above the respective local normative reference range. This group underwent further clinical phenotyping by retrospective electronic case note review for age, menopausal status, body mass index, ethnicity, clinical presentation, and the underlying cause of androgen excess. The underlying diagnosis was supported by clinical, biochemical, and radiological findings in each case, with final review of the diagnosis by two certified endocrinologists (M.W.O. and W.A.). The diagnosis of PCOS was established according to Rotterdam criteria (1).

### Serum androgen measurements

Biochemical androgen excess for each metabolite was defined as a serum concentration increased above the laboratory and age-specific normative reference range. Serum A4 and T were analyzed by liquid chromatography-tandem mass spectrometry on a Prominence XR UPLC (Shimadzu Corporation) coupled to a Triple Quad 6500 mass spectrometer (SCIEX). Briefly, samples are analyzed by liquid/liquid extraction following addition of deuterated internal standards (T-d3 and A4-d7; QMX Laboratories). Samples were separated chromatographically using an isocratic elution profile, ionized using positive atmospheric pressure chemical ionization, and detected using the following transitions: T, 289 > 109 and 289 > 97; T-d3, 292 > 97; A4, 287 > 109 and 287 > 97; and A4-d7, 294 > 100. The measuring range for serum T was 0.1 to 80 nmol/L and for serum A4 0.3 to 80 nmol/L. The coefficients of variation for T and A4 were 5.5% to 17% and 6.1% to 14%, respectively. Serum DHEAS was analyzed using the competitive electrochemiluminesence immunoassay on the Cobas c702 analyzer (Roche); the corresponding measuring range was 0.01 to 27.00 µmol/L and the coefficient of variation range 3.2% to 5.0%.

We arbitrarily defined the severity of androgen excess as three severity levels: mild, intermediate, and severe, separately for premenopausal and postmenopausal women (Table 1). This was done by prior agreement (W.A. and M.W.O.) based on clinical experience with knowledge of the local reference ranges but blinded to the results of androgen measurements in the cohort.

**Table 1.** Severity Levels of Androgen Excess for Serum DHEAS, A4, and T

Serum Androgen Status	Serum DHEAS (μmol/L)	Serum A4 (nmol/L)	Serum T (nmol/L)
Normal reference range			
Premenopause (<50 y)	0.92–7.6	0.9–7.5	<1.9
Postmenopause (>50 y)	0.26–5.5	0.4–2.9	<1.9
Mild androgen excess			
Premenopause (<50 y)	7.7–12.5	7.6–11.5	1.9–2.9
Postmenopause (>50 y)	5.6–12.5	3.0–7.9	1.9–2.9
Intermediate androgen excess			
Premenopause (<50 y)	12.6–19.9	11.6–16.5	3.0–4.9
Postmenopause (>50 y)	12.6–19.9	8.0–12.9	3.0–4.9
Severe androgen excess			
Premenopause (<50 y)	≥20.0	>16.5	≥5.0
Postmenopause (>50 y)	≥20.0	≥13.0	≥5.0

The three levels (mild, intermediate, and severe) were arbitrarily defined, with separate cutoffs for premenopausal and postmenopausal women. Normal reference ranges (5th to 95th centile) were defined for serum A4 and T (measured by tandem mass spectrometry) in a female local reference population (n = 355) and for serum DHEAS (measured by immunoassay) referring to a female reference population (n = 516) provided by the manufacturer.

### Statistical analysis

SPSS version 22 (SPSS Inc.) was used for descriptive data analysis. Where relevant, data are expressed as median and first and third quartile, unless otherwise stated. The Mann-Whitney *U* test was used for comparison between two groups (premenopausal and postmenopausal). Statistical significance was set at *P* < 0.05.

## Results

### Description of the cohort and diagnostic spectrum

**Figure 1.**
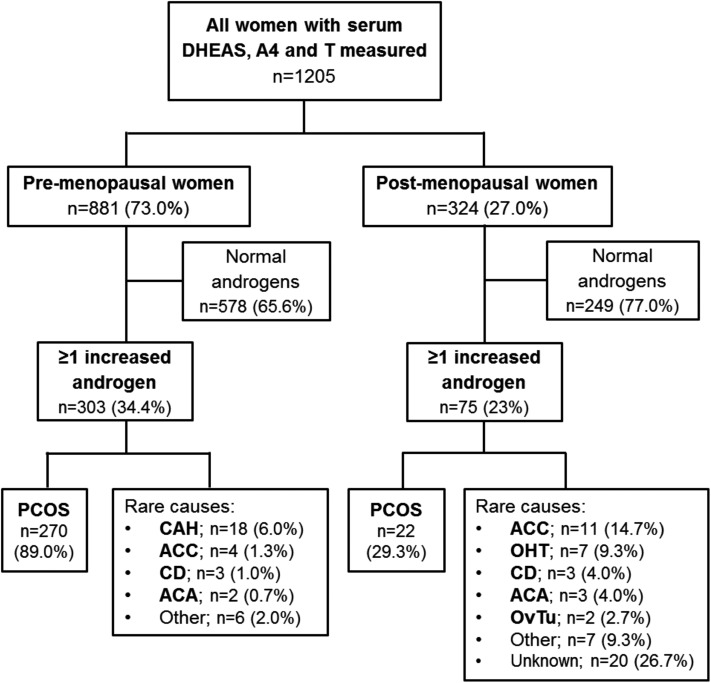
Flow chart of distribution of diagnoses according to premenopausal vs postmenopausal status in 1205 women who underwent simultaneous measurement of DHEAS, A4, and T. OvTu, ovarian tumor.

A total of 2269 women had at least one serum concentration of DHEAS, A4, or T measured during the study period. In 1064 women (46.9%), only one or two of the three androgens had been measured, and increased concentrations for serum DHEAS, A4, and T were found in 13.3%, 12.8%, and 13.0%, respectively. In 1205 women (53.1%), all three androgens had been measured simultaneously, indicative of a higher clinical suspicion of androgen excess (Fig. 1). The majority of these patients were premenopausal (n = 881; 73.1%).

Overall, 378 of the 1205 women (31.4%) had increased serum concentrations of one or more of DHEAS, A4, and T and underwent clinical phenotyping, as described in the *Methods* section. Of the 378 women with androgen excess, 303 (80.2%) were premenopausal; the median age in the premenopausal group was 29 years compared with 62 years in the postmenopausal group (Supplemental Table 1). The majority of women in both groups were of Caucasian ethnicity. Although a total of 87 (28.7%) premenopausal women were of South Asian ethnicity, only 6 (8.0%) South Asian women were identified in the postmenopausal group. Median body mass index (range) was 30.0 kg/m^2^ (16 to 65) and 32.0 kg/m^2^ (15 to 47) in premenopausal and postmenopausal women, respectively.

In premenopausal women, irregular or absent menstrual periods and clinical signs of androgen excess were the most common causes for assessment of androgens (47.0% and 48.0%, respectively), followed by difficulties to conceive (5.5%). In postmenopausal women, clinical hyperandrogenism was the most frequent cause leading to measurement of serum androgens (57.0%). Rare causes included overt virilization (0.5% in premenopausal and 7.0% in postmenopausal) and adrenal incidentaloma (0.5% in premenopausal and 12.0% of postmenopausal).

Although the majority of the 378 women with at least one increased serum androgen had an underlying diagnosis of PCOS (77.2%), rare and very rare causes of androgen excess were also identified, including congenital adrenal hyperplasia (CAH; 4.8%), adrenocortical carcinoma (ACC; 4.0%), ovarian hyperthecosis (OHT; 1.9%), Cushing disease (CD; 1.6%), adrenocortical adenoma (ACA; 1.3%), and ovarian tumors (0.5%). In 36 patients (9.5%), no explanation for the increased serum androgen concentrations was identified. PCOS was the most common diagnosis in both premenopausal and postmenopausal women; however, the number of PCOS diagnoses was much lower in the postmenopausal group (29.3% vs 89.0%). Among all women with PCOS (n = 292), 266 (91.1%) were not taking antiandrogenic medication or metformin at the time of biochemical assessment, 7 (2.4%) were on a contraceptive pill, 3 (1.1%) took spironolactone, and 16 (5.4%) metformin.

The next most common diagnosis in premenopausal women was CAH (n = 18; 6.0%), whereas in postmenopausal women, ACC (n = 11; 14.7%) and OHT (n = 7; 9.3%) were most prevalent after PCOS (Fig. 1). Of the 18 patients with CAH, 2 were newly diagnosed and untreated at the time of androgen measurement, whereas the remaining 16 had a previous diagnosis of CAH but had been reviewed at our institution for either a second opinion and advice on difficult-to-control disease or because they moved into the catchment area of our hospital.

Median (range) sex hormone-binding globulin levels (nmol/L) in patients with PCOS, CAH, ACC, and OHT were 37.7 (11 to 212.2), 44.5 (21 to 131.1), 64.2 (28.8 to 270), and 60.3 (38.8 to 218.3), respectively; the corresponding free androgen indexes were determined as 4.5 (0.3 to 49.6), 8.7 (1.4 to 117.9), 8.4 (0.6 to 39.2), and 10.6 (4.4 to 28.4), respectively.

### Patterns of androgen excess according to the underlying diagnosis

**Figure 2.**
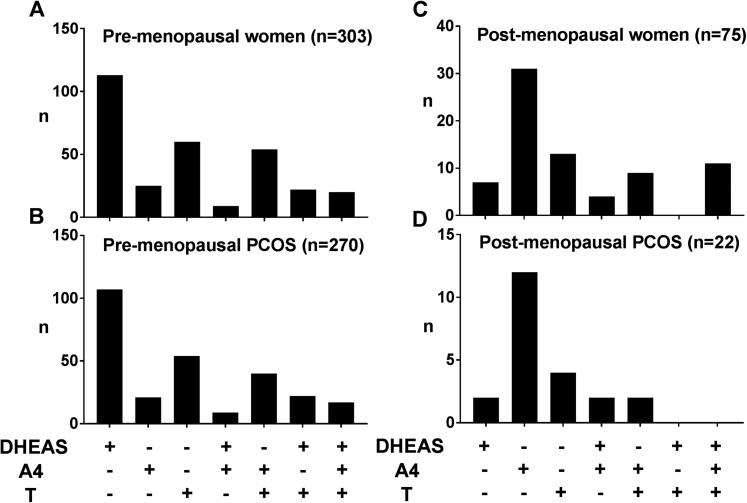
Distribution of androgen excess patterns in (A) all premenopausal women, (B) premenopausal women with underlying PCOS, (C) all postmenopausal women, and (D) postmenopausal women with underlying PCOS. +, increased; −, not increased.

In the premenopausal group, increased serum DHEAS with normal A4 and normal T was the most frequent pattern observed (37.3%); an isolated increase in A4 was the most frequent pattern observed in the postmenopausal cohort (41.3%) (Fig. 2A and 2B). Mirroring this, increased serum DHEAS was the most common constellation in the premenopausal women with PCOS, whereas isolated increased A4 was most commonly found in postmenopausal women with PCOS (Fig. 2C and 2D).

**Figure 3.**
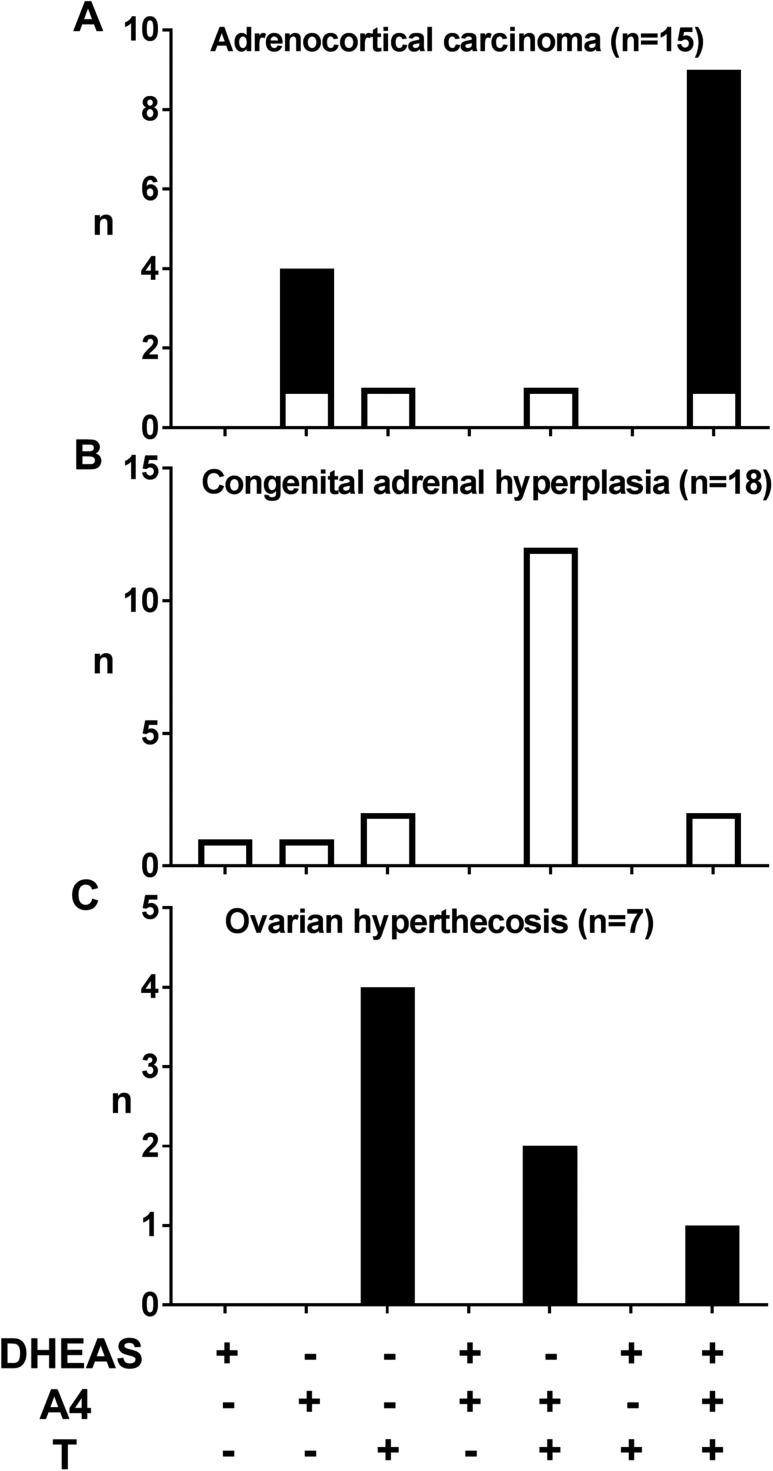
Distribution of androgen excess patterns in (A) ACC, (B) CAH, and (C) OHT. +, increased; −, not increased; black bars, postmenopausal; white bars, premenopausal women.

In the postmenopausal group, 14.8% of women had all three serum androgens increased. This pattern was not observed in any postmenopausal woman with a diagnosis of PCOS (Fig. 2B–2D) but most commonly found in postmenopausal cases of ACC and in some women with CAH and OHT (Fig. 3). Although A4 was increased in all postmenopausal cases of ACC (n = 11; Fig. 3A), there was no consistent pattern of androgen excess in premenopausal women with ACC (n = 4; Fig. 3A). In women with CAH, all of whom were premenopausal, the combination of increased T and A4 was the most frequent pattern observed (n = 12) (Fig. 3B). All cases of OHT, only diagnosed in postmenopausal women, had increased serum T and in four of them in isolation (n = 7; Fig. 3C).

### Severity of androgen excess

Increased serum androgen concentrations were arbitrarily divided into three levels of severity (mild, intermediate, and severe; see Methods and Supplemental Tables 2 and 3).

**Figure 4.**
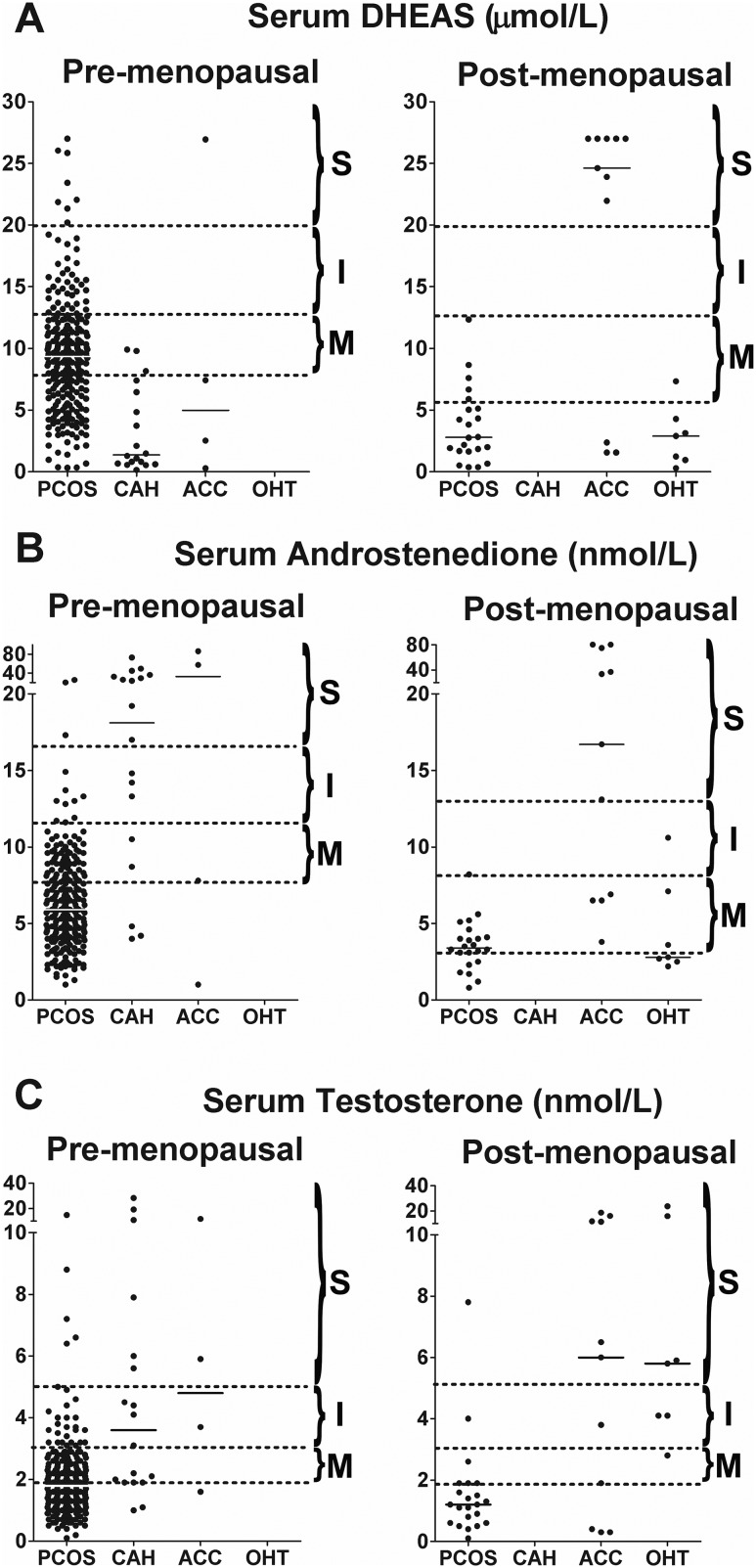
Severity of androgen excess according to diagnosis and androgen measured. Three levels of androgen excess [mild (M), intermediate (I), and severe (S)] were arbitrarily defined for each androgen (for cutoffs, see Table 1) and are demarcated by dotted lines. Median values for each diagnosis are denoted by a solid black line.

In premenopausal women, any level of DHEAS excess was overwhelmingly caused by PCOS (Fig. 4A). CAH was commonly found in premenopausal women with severe A4 excess (59%; intermediate, 25%; and mild, 2.5%) and T excess (43%; intermediate, 14.3%; and mild, 5.3%). DHEAS excess in CAH was less frequent and invariably mild (Fig. 4A–4C). There were only four cases of ACC among the 303 premenopausal women; all affected women showed severe androgen excess, including one or more of the three analytes.

In postmenopausal women with PCOS, intermediate or severe excess of any androgen was rare (Fig. 4A–4C). Severe DHEAS excess was exclusively observed in ACC, whereas DHEAS excess in PCOS or OHT was rare and, if present, only mild (Fig. 4A). Similarly, severe A4 excess was exclusively found in ACC (Fig. 4B); mild A4 excess was present in 68.2% of the postmenopausal PCOS cases, but 14.2% of women with mild A4 excess were diagnosed with ACC or OHT. Severe T excess was either due to ACC or OHT, with a single case of PCOS (Fig. 4C); in postmenopausal women with mild and intermediate T excess, we found similar numbers of cases of ACC, OHT, and PCOS.

Causes of non-PCOS–related androgen excess even rarer than CAH, ACC, and OHT were not reported frequently enough to discern distinct patterns (see Supplemental Tables 2 and 3 for findings in the premenopausal and postmenopausal groups, respectively).

## Discussion

Our study assessed simultaneously measured serum concentrations of the key androgen precursors DHEAS and A4 and the active androgen T in 1205 consecutively recruited women, which represents the largest systematically recruited androgen excess cohort reported to date and the only to include a large subgroup of postmenopausal women. This has enabled us to provide an age group–specific analysis of differences in pattern and severity of androgen excess and their relation to underlying pathologies, with the description of distinct androgen excess signatures that have the potential to guide further diagnostic workup.

A number of previous studies have assessed the prevalence of androgen excess and underlying non-PCOS pathology in cohorts of >100 women referred to secondary care with clinical features of hyperandrogenism (see summary of studies in Table 2) (21–29). We identified a total of 9 studies with a median of 318 patients (range 152 to 950). These studies recruited overwhelmingly premenopausal women, with several measuring serum T and DHEAS but not A4, including the two largest studies by Azziz *et al.* (26) and Carmina *et al.* (25) of 873 and 950 women, respectively. The majority of these women had an underlying diagnosis of PCOS or idiopathic hirsutism. All previous studies taken together have collectively detected eight cases of androgen-producing ovarian tumor, four cases of ACC, and two cases of CD. Of note, these studies included a large proportion of women recruited from gynecologic and reproductive endocrine clinics as opposed to from across the whole range of a specialist endocrine referral center, as in our study, which is likely to lead to the detection of greater proportion of disorders other than PCOS.

**Table 2.** Summary of Previously Published Studies That Aimed to Define the Frequency of Disorders Observed in Women Who Had Serum Androgens Measured for Clinically Suspected Androgen Excess

First Author (Reference Number)	Year of Publication	n	Menopausal Status	Setting	Presentation	Androgens Measured (Method)	PCOS, n (%)	Idiopathic Hirsutism, n (%)	CAH, n (%)	Androgen-Secreting Tumor, n (%)	Other, n (%)
Di Fede (21)	2010	152	Premenopausal	Endocrinology department	Mild hirsutism (Ferriman–Gallwey score 8–15)	DHEAS, T, and A4 (immunoassay)	72 (47.4)	77 (50.6)	3 (2.0)	—	—
Karrer-Voegeli (22)	2009	318	Premenopausal	Endocrinology department	Skin manifestations of androgen excess	DHEAS, T, and A4 (immunoassay)	62 (27.6)	—	4 (1.8)	OvTu, 2 (0.9)	Idiopathic hyperandrogenism (hirsutism or elevated androgens), 101 (44.3)
Escobar-Morreale (23)	2008	270	Premenopausal	Endocrinology department	Oligo-/amenorrhea and/or skin manifestations of androgen excess	DHEAS, T, and A4 (immunoassay)	171 (63.3)	24 (8.9)	6 (2.2)	—	Idiopathic hyperandrogenism, 61 (22.6); hyperprolactinemia, 2 (0.7%)
Fanta (24)	2008	298	Premenopausal	Obstetrics and gynecology department	Oligo-/amenorrhea and/or skin manifestations of androgen excess and biochemical androgen excess	DHEAS, T, and A4 (immunoassay)	290 (97.3)	—	8 (2.7)	—	—
Carmina (25)	2006	950	Premenopausal	Two endocrinology departments	Skin manifestations of androgen excess	DHEAS and T (immunoassay)	685 (72.1)	72 (7.6)	41 (4.3)	OvTu, 2 (0.2)	Idiopathic hyperandrogenism, 150 (15.8)
Azziz (26)	2004	873	Premenopausal	Obstetrics and gynecology reproductive endocrinology department	Oligo-/amenorrhea and/or skin manifestations of androgen excess	DHEAS and T (immunoassay)	716 (82.0)	39 (4.7)	24 (2.2)	OvTu, 2 (0.2)	Hyperandrogenic insulin-resistant acanthosis nigricans, 33 (3.1)
Glintborg (27)	2004	340	Premenopausal	Endocrinology department	Hirsutism	DHEAS and T (immunoassay)	134 (39.4)	201 (59.1)	2 (0.6)	OvTu, 1 (0.3)	Prolactinoma, 1 (0.3)
											CD, 1 (0.3)
Unluhizarci (28)	2004	168	Premenopausal	Endocrinology department	Hirsutism	DHEAS, T, and A4 (immunoassay)	96 (57.1)	27 (16.0)	12 (7.1)	ACC, 3 (1.8)	CD, 1 (0.6)
O'Driscoll (29)	1994	350	Premenopausal and postmenopausal (322 and 28 women, respectively)	Endocrinology department	Skin manifestations of androgen excess	DHEAS, T, and A4 (immunoassay)	170 (60.0) of the 282 women who had an ultrasound scan	—	3 (0.8)	ACC, 1 (0.2)	Cortisone reductase deficiency, 1 (0.2)
							Two (12.0) of the 17 postmenopausal women who were scanned had PCO*^a^*			OvTu, 1 (0.2)	

Only studies with >100 patients were included.

Abbreviation: OvTu, ovarian tumor.

^a^ Diagnosis of PCOS in this study was based on the demonstration of polycystic ovaries on ultrasound.

Our study has included all patients in whom serum androgen measurements were requested by any endocrinologist across a large secondary/tertiary care specialist service during a 5-year period. This included patients presenting with clinical features of hyperandrogenism, but also patients assessed for suspected or confirmed endocrine conditions that may be associated with androgen excess, such as adrenal masses or Cushing syndrome, resulting in higher complexity and diversity of the underlying diagnostic spectrum. This recruitment pattern will have contributed to a substantial number of cases with non-PCOS–related causes of androgen excess in our cohort, but may also have resulted in a relevant proportion of postmenopausal women. Our recruitment was very much reflective of the ethnic makeup of the larger region we recruited from (30), comprising ∼10% of the United Kingdom population. The only exception was a lower than expected number of South Asian women in the postmenopausal age group, potentially indicative of difficulties in accessing health care in this group of older women who often are non-English speaking.

Unsurprisingly, PCOS was the most prevalent cause of androgen excess in the premenopausal cohort and in one-third of postmenopausal women in our study. Although the measurement of serum T is traditionally regarded as a sensible first-line biomarker of circulating androgen burden in women (31), our data support that simultaneous measurement of DHEAS and A4 alongside T increases the sensitivity for detection of PCOS-related androgen excess. Employing concurrent measurement of DHEAS, A4, and T, our study demonstrated that only 49% of premenopausal and 27% of postmenopausal women with PCOS would have been detected by isolated measurement of serum T, currently recommended as the single diagnostic androgen marker for the diagnosis of PCOS (32). Our findings corroborate previous reports from our group and subsequently others that A4 is a more sensitive marker of PCOS-related androgen excess than serum T (4, 33), which has been recognized in the recent European position statement on PCOS (31). Increased serum DHEAS concentrations were the most prevalent finding in the premenopausal women with PCOS in our study, further highlighting the well-reported significance of serum DHEAS as a major marker of PCOS (34–36). In our postmenopausal PCOS cases, serum A4 was the primary androgen marker of PCOS. This shift in androgen excess pattern at menopause may be reflective of the age-related decline in adrenal DHEAS production, the so-called adrenopause, which occurs alongside the menopausal transition (37).

Importantly, we could show that the concurrent measurement of all three androgens and subsequent analysis of pattern and severity of androgen excess provide important clues for the detection of ovarian and adrenal neoplastic disease and other rare causes of androgen excess that may require urgent diagnostic and therapeutic management. Most frequently observed causes of non-PCOS androgen excess were CAH and ACC in premenopausal women and ACC and OHT in women of postmenopausal age. CAH was the most prevalent cause in premenopausal women, with most patients showing combined increases in A4 and T. Disproportionate elevations of A4 in comparison with T in women with CAH is typically a marker of poor disease control, and the majority of increased circulating T in CAH is due to peripheral activation from A4, rather than direct adrenal overproduction (38). Although ACC was rare in premenopausal women (1.3%), there was a relevant number of postmenopausal ACC cases (14.7%). Nearly all postmenopausal cases of ACC had increased A4, and the majority had combined increases of DHEAS, A4, and T. Only 60% of patients with ACC had increased DHEAS, the steroid most often recommended as a biochemical screen for ACC in large adrenal tumors, adding weight to the previous recommendation to always measure all three androgens in tandem in suspected ACC (39). In postmenopausal women, severe levels of DHEAS and A4 excess were only observed in ACC, whereas severe T excess was equally observed in ACC and OHT, which was the second most common non-PCOS–related cause of androgen excess in the postmenopausal age group (9.3%). All cases of OHT had increased T, often in combination with A4 and DHEAS, but severe excess was only observed for serum T.

Previous publications in this area have almost exclusively reported immunoassays for androgen measurement, whereas current progress in androgen analysis by mass spectrometry offers the opportunity for simultaneous multisteroid profiling. Our study used tandem mass spectrometry for the combined highly sensitive and specific measurement of A4 and T. The direction of travel in clinical biochemistry is clearly toward the development of even more wide-ranging multisteroid profiling mass spectrometry assays, often confronting the biochemist and clinician with increased analytes that were not specifically requested by the clinician but are automatically measured by the assay. Our findings can serve as some guide for risk stratification in the situation of incidental androgen excess findings. In addition to the classic androgen synthesis pathway from DHEAS via A4 to T and DHT, recent studies have highlighted the significance of the 11-oxygenated androgen pathway (40, 41). This is initiated by conversion of A4 to 11-hydroxyandrostenedione by adrenal CYP11B1, with further downstream activation to 11keto-testosterone and 11keto-dihydrotestosterone, which bind and activate the androgen receptor with similar affinity and potency as T and DHT. Our own work and that of others has underlined the 11-oxygenated androgens as the major circulating androgens in PCOS (42) and important androgen excess parameters in CAH (43–45). In this study, we did not measure 11-oxygenated androgens, as the serum samples were analyzed in the routine clinical biochemistry setting where this assay is not yet available. However, it will be very interesting to analyze the impact of 11-oxygenated androgen measurement on further delineation of condition-specific androgen excess signatures.

A limitation of the study is in the definition of premenopausal and postmenopausal by an age cutoff of 50 years. However, we established the menopausal status in the 378 women with increased androgens as part of the clinical data extraction. Among the 303 women aged <50 years, only 2 (0.6%) had age-related ovarian failure with increased gonadotropins. Similarly, only 3 (4%) of the 75 women aged >50 years had persistent menses. We have applied the age cutoff of 50 years to stratify the 827 women with normal androgen results. This enabled us to calculate the proportions of women investigated and of those with abnormal results (Fig. 1). Other limitations of our study include the arbitrary definition of the severity cutoffs and the population bias, as we recruited from a secondary/tertiary care cohort enriched for patients with endocrine disorders associated with androgen excess other than PCOS, which, however, enabled us to analyze a relevant number of cases and make condition-specific observations on androgen excess pattern and severity.

In conclusion, we have undertaken a detailed analysis of a large consecutive cohort of women undergoing serum androgen measurement for suspected androgen excess, providing unique insights into the diagnostic utility of combined measurement of serum DHEAS, A4, and T concentrations. Thereby, our findings may help to rationalize the need for further investigations such as imaging or functional testing with dexamethasone and gonadotropin-releasing hormone superagonists for adrenal and ovarian androgen suppression, respectively. The results of this study highlight the importance of considering patient age and the severity of biochemical disturbances as predictors of underlying non-PCOS pathology. In premenopausal women, severe androgen excess should significantly increase the clinical suspicion of non-PCOS pathology, whereas after the age of 50, even intermediate androgen excess should prompt further investigations.

## Supplementary Material

Supplemental Table 1

## Abbreviations:

A4: androstenedione
CAH: congenital adrenal hyperplasia
CD: Cushing disease
DHEA: dehydroepiandrosterone
DHEAS: dehydroepiandrosterone sulfate
DHT: 5*α*-dihydrotestosterone
OHT: ovarian hyperthecosis
PCOS: polycystic ovary syndrome
T: testosterone
