# Supplementary material for: Causes, Patterns, and Severity of Androgen Excess in 1205 Consecutively Recruited Women
Source: J Clin Endocrinol Metab. 2018 Jan 12;103(3):1214–23. doi: 10.1210/jc.2017-02426 (PMC5868408; doi:10.1210/jc.2017-02426)
Supplement: Supplemental Table 1 [file jc.2017-02426_supplemental_table_1.docx]

**Supplemental Table 1: Baseline demographics of 378 women with biochemical androgen excess**. All had at least one of serum DHEAS, androstenedione and testosterone increased above the reference range and were derived from a cohort of 1205 patients with all three androgens measured. Data are presented as median (first quartile (Q1), third quartile (Q3)) where appropriate.

|  | **Total** | **Premenopausal** | **Postmenopausal** |
| --- | --- | --- | --- |
| **Patients with ≥1 of 3 androgens increased** | 378 | 303 (80·2%) | 75 (19·8%) |
| **Age (years)**  Median (Q1, Q3) | 29 (22, 43) | 26 (21, 32) | 62 (55, 70) |
| **BMI (kg/m^2^)**  Median (Q1, Q3) | 30 (25, 37) | 30 (24, 37) | 32 (26, 36) |
| **Ethnicity***   - **Caucasian** | 264 (69·8%) | 196 (64·7%) | 68 (90·7%) |
| - **South Asian** | 93 (24·6%) | 87 (28·7%) | 6 (8·0%) |
| - **Afro-Caribbean** | 7 (1·9%) | 6 (2·0%) | 1 (1·3%) |
| - **Mixed background** | 6 (1·6%) | 6 (2·0%) | 0 |
| - **Other** | 2 (0·5%) | 2 (0·6%) | 0 |
| - **Unknown** | 6 (1·6%) | 6 (2·0%) | 0 |

* Ethnicity distribution pattern in the Birmingham area is: Caucasians 58·0%, Asians 26·6% (South Asians 22·5%, other Asians 4·08%), Afro-Caribbeans 9·0%, Mixed 4·4% and other 2·0%. Source: United Kingdom Census 2011, Office for National Statistics.

**Supplemental Table 2**: **Severity levels of androgen excess in premenopausal women according to diagnosis (n; % of condition)**. For the specific cut-offs relating to the three severity levels (mild, intermediate and severe), please see **Table 1** in the main paper. PCOS, polycystic ovary syndrome; CAH, congenital adrenal hyperplasia; ACC, adrenocortical carcinoma; CD, Cushing’s disease; ACA, adrenocortical adenoma.

| Total  n=303 | **PCOS**  n=270 | **CAH**  n=18 | **ACC**  n=4 | **CD**  n=3 | **ACA**  n=2 | **Other***  n=6 |
| --- | --- | --- | --- | --- | --- | --- |
| **DHEAS** (μmol/L) | | | | | | |
| Normal  (n=114) | 91  34% | 15  83% | 3  75% | 2  66% | 1  50% | 2  33% |
| Mild  (n=138) | 131  48% | 3  17% | - | - | 1  50% | 3  50% |
| Intermediate  (n=41) | 40  15% | - | - | 1  33% | - | - |
| Severe  (n=10) | 8  3% | - | 1  25% | - | - | 1  17% |
| **Androstenedione** (nmol/L) | | | | | | |
| Normal  (n=195) | 183  68% | 3  17% | 1  25% | 1  33% | 1  50% | 6  100% |
| Mild  (n=79) | 75  28% | 2  11% | 1  25% | 1  33% | - | - |
| Intermediate  (n=12) | 9  3% | 3  17% | - | - | - | - |
| Severe  (n=17) | 3  1% | 10  55% | 2  50% | 1  33% | 1  50% | - |
| **Testosterone** (nmol/L) | | | | | | |
| Normal  (n=147) | 137  45% | 2  11% | 1  25% | 2  67% | 2  100% | 3  50% |
| Mild  (n=113) | 104  34% | 6  33% | - | 1  33% | - | 3  50% |
| Intermediate  (n=28) | 23  9% | 4  22% | 1  25% | - | - | - |
| Severe  (n=14) | 6  2% | 6  33% | 2  50% | - | - | - |

* Three cases of DHEA over-replacement in adrenal insufficiency and three cases of mildly raised testosterone likely secondary to raised sex hormone binding globulin (SHBG).

**Supplemental Table 2**: **Severity levels of androgen excess in postmenopausal women according to diagnosis (n; % of condition)**. For the specific cut-offs relating to the three severity levels (mild, intermediate and severe), please see **Table 1** in the main paper. PCOS, polycystic ovary syndrome; ACC, adrenocortical carcinoma; OHT, ovarian hyperthecosis; CD, Cushing’s disease; ACA, adrenocortical adenoma; OvTu, ovarian tumour

| Total  n=75 | **PCOS**  n=22 | **ACC**  n=11 | **OHT**  n=7 | **CD**  n=3 | **ACA**  n=3 | **OvTu**  n=2 | **Other***  n=7 | **Unknown** n=20 |
| --- | --- | --- | --- | --- | --- | --- | --- | --- |
| **DHEAS** (μmol/L) | | | | | | | | |
| Normal  (n=54) | 17  77% | 3  27% | 6  86% | 3  100% | 2  67% | 2  100·0% | 2  29% | 19  95% |
| Mild  (n=11) | 5  23% | - | 1 14% | - | 1  33% | - | 3  42% | 1  5% |
| Intermediate  (n=2) | - | - | - | - | - | - | 2  29 | - |
| Severe  (n=8) | - | 8  73% | - | - | - | - | - | - |
| **Androstenedione** (nmol/L) | | | | | | | | |
| Normal  (n=19) | 6  27% | - | 4  57% | - | 2  67% | 1  50% | 3  43% | 3  15% |
| Mild  (n=42) | 15  68% | 4  36% | 2  29% | 1  33% | 1  33% | 1  50% | 4  57% | 16  80% |
| Intermediate  (n=4) | 1  5% | - | 1  14% | 1  33% | - | - | - | 1  5% |
| Severe  (n=8) | - | 7  64% | - | 1  33% | - | - | - | - |
| **Testosterone** (nmol/L) | | | | | | | | |
| Normal  (n=42) | 16  72% | 3  27% | - | 2  67% | 1  33% | - | 3  43% | 17  85% |
| Mild  (n=14) | 4  18% | 1  9%1 | 1  14% | 1  33% | 1  33% | - | 3  43% | 3  15% |
| Intermediate  (n=5) | 1  5% | 1  9% | 2  29% | - | - | - | 1  14% | - |
| Severe  (n=14) | 1  5% | 6  55% | 4  57% | - | 1  33% | 2  100% | - | - |

*Five cases of DHEA over-replacement in adrenal insufficiency and two cases of mildly raised testosterone likely secondary to raised sex hormone binding globulin (SHBG).


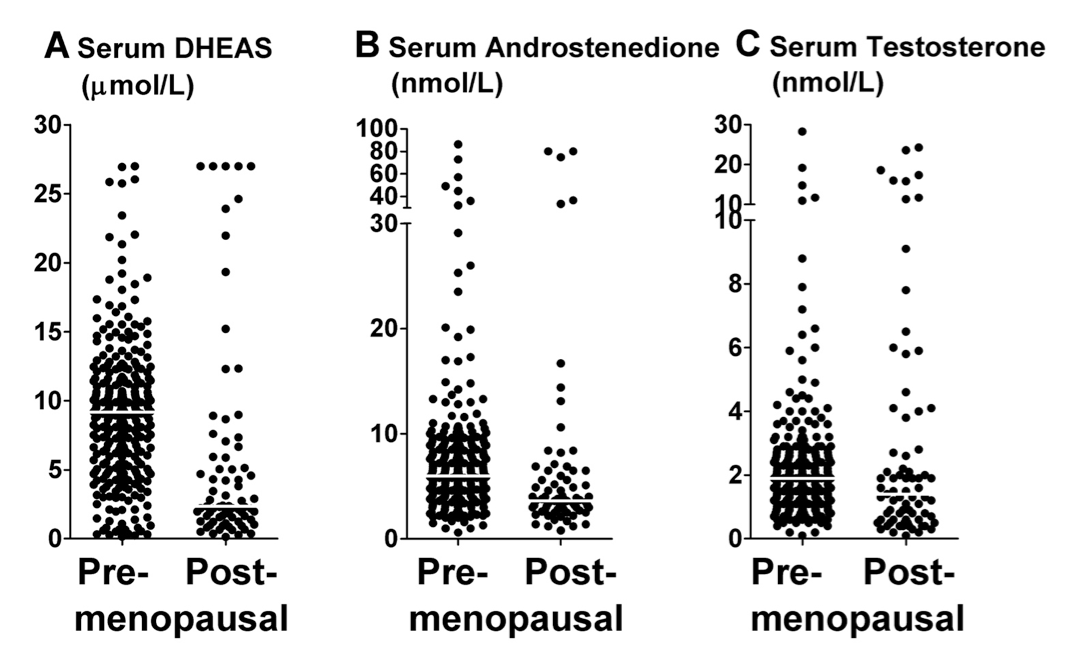


**Suppl. Fig. 1**: Concentrations of serum concentrations of (A) DHEAS, (B) androstenedione and (C) testosterone in premenopausal compared to postmenopausal women of our cohort. Median values for each group are denoted by a solid white line.
